# Altered Functional Connectivity in the Resting State Neostriatum After Complete Sleep Deprivation: Impairment of Motor Control and Regulatory Network

**DOI:** 10.3389/fnins.2021.665687

**Published:** 2021-08-17

**Authors:** Haiteng Wang, Ke Yu, Tianyi Yang, Lingjing Zeng, Jialu Li, Cimin Dai, Ziyi Peng, Yongcong Shao, Weiwei Fu, Jianlin Qi

**Affiliations:** ^1^School of Psychology, Beijing Sport University, Beijing, China; ^2^Department of Neurology, The General Hospital of Western Theater Command, Chengdu, China; ^3^Suzhou Institute of Biomedical Engineering and Technology, Chinese Academy of Sciences, Suzhou, China; ^4^Air Force Medical Center, Beijing, China

**Keywords:** sleep deprivation, putamen, caudate, resting state-fMRI, functional connectivity, motor control network

## Abstract

Sleep loss not only compromises individual physiological functions but also induces a psychocognitive decline and even impairs the motor control and regulatory network. In this study, we analyzed whole-brain functional connectivity changes in the putamen and caudate nucleus as seed points in the neostriatum after 36 h of complete sleep deprivation in 30 healthy adult men by resting state functional magnetic resonance imaging to investigate the physiological mechanisms involved in impaired motor control and regulatory network in individuals in the sleep-deprived state. The functional connectivity between the putamen and the bilateral precentral, postcentral, superior temporal, and middle temporal gyrus, and the left caudate nucleus and the postcentral and inferior temporal gyrus were significantly reduced after 36 h of total sleep deprivation. This may contribute to impaired motor perception, fine motor control, and speech motor control in individuals. It may also provide some evidence for neurophysiological changes in the brain in the sleep-deprived state and shed new light on the study of the neostriatum in the basal ganglia.

## Introduction

Total sleep deprivation (TSD) refers to a physiological state of less than 4 h of continuous sleep for at least 24 h. TSD has been shown to not only harm individual physiological functions and increase the risk of developing cardiovascular disease and obesity (St-Onge and Zuraikat, 2019; Yu et al., 2020) but also cause a psychocognitive decline including loss of mood, learning, and memory, which in turn triggers individual behavioral disorders and can even cause operational accidents (Killgore, 2010; Tantawy et al., 2013; Goldstein and Walker, 2014; Xie et al., 2015; Lo et al., 2016; Krause et al., 2017; Cunningham et al., 2018; Peng et al., 2020).

Accidents are associated not only with psychocognitive decline but also with impaired fine motor control. Fine motor control is a high-level cognitive function of humans, which belongs to the category of voluntary movement, and occurs throughout the whole process of human daily life and social activities, such as reading, writing, speech, working, and some sports (Stippich et al., 2007; Bracci et al., 2012; Hao et al., 2016). Because fine movement is very important in daily life, much research has been conducted in the field of cognitive neuroscience, applying anatomical, physiological, and molecular biological methods to study voluntary movement and the mechanisms that govern it. The basic anatomical structures, physiological functions, and interconnections among various structures involved in voluntary motor control in animals and humans have now been established. In recent years, with the rapid development of medical imaging, functional magnetic resonance imaging (fMRI) techniques have provided further insights into the *in vivo* study of neural mechanisms, as well as into the imaging mechanism of fine motor control (Lee et al., 2010; Plow et al., 2010). Resting-state functional MRI (rs-fMRI) reflects spontaneous activity *via* the blood oxygenation level dependent (BOLD) signal in the brain, which is closer to the physiological state. Relative to the task state, rs-fMRI is convenient to operate, provides repetitive, stable, and reliable information, and can be analyzed in many ways. Therefore, rs-fMRI has obvious advantages for studying brain spontaneous activity, functional connectivity among various brain regions, development and plasticity of brain function, and neuropsychiatric disorders, and is a recent focus of research on brain function (Friston, 2004; Zhu et al., 2020).

In general, voluntary motor signals emanate from the area of the precentral gyrus of the cerebral cortex through descending pyramidal system conduction pathways to the anterior horn of the spinal cord and then through spinal nerves to the corresponding motor neurons, causing skeletal muscle contraction. The precentral gyrus plays an important role in voluntary movement of the soma and is a somatomotor high-level control center. Multiple precentral gyrus regions are involved in composing the somatomotor cortex, including the primary motor cortex (M1), premotor area (PM), and supplementary motor area (SMA), which are located within and lateral to the brain in the precentral gyrus. These different regions of the precentral gyrus play different roles in the achievement of motor function, acting in the form of network connections (Mayka et al., 2006). However, the brain regions involved in motor control function are extensive, and voluntary motor information undergoes regulatory control by the extrapyramidal system in addition to the pyramidal system. These include the spinal cord, thalamus, secondary somatosensory cortex (Supplementary Table 2), medial insular cortex (IC), anterior cingulate cortex (ACC), PM, SMA, and M1. Furthermore, the limbic system, basal ganglia (BG), thalamus, orbitofrontal cortex (OFC), prefrontal cortex (PFC), ACC, PM, SMA, and M1 regions make up the locomotor modulatory excitatory pathway (Tanaka and Watanabe, 2012).

However, because of limitations in research conditions, the mechanisms of motor control involved in the basal ganglia have not been thoroughly elucidated regarding the initiation, programming of voluntary movement, and the execution of movement in humans. The BG are important nuclei in extrapyramidal transmission pathways that receive afferent signals from the cortex to feed back to the cerebral cortex after integration. From a functional point of view, the BG can be divided into six functional nuclei, including the striatum (STR), external globus pallidus (GPe), internal segment of the globus pallidus, substantia nigra pars reticulata (GPi-SNr), substantia nigra pars compacta (SNc), and subthalamic nucleus (STN) (Rodriguez-Sabate et al., 2016).

In recent years, functional connectivity analyses have provided invaluable approaches for studying the human brain on the brain-network level. Using fMRI approaches, investigators have found specific patterns of functional connectivity in the sensorimotor network between the BG and the primary motor cortex and cerebellum (Carlson, 2009; Sokolov et al., 2012), in which the STR, together with the STN and substantia nigra, mainly constitute the subcortical circuits regulating locomotion, in concert with the cerebral cortex and cerebellum regulating voluntary movements, muscle tone, and postural reflexes (Wall et al., 2013). Therefore, the BG play a very important role in the sensorimotor network and mainly participate in the motion control and regulation network (Bostan et al., 2013; Zhang et al., 2018) responsible for physiological functions such as motor control, motor learning, functioning, and behavior (Lanciego et al., 2012).

Among the anatomical structures of the BG, the STR includes the caudate and lentiform nucleus, which are connected anteriorly ventrally; the lentiform nucleus is further divided into the putamen and the pallidus; the caudate nucleus and putamen are phylogenetically more recent structures of the STR, together called the neostriatum, and the pallidus is the oldest part of the STR, called the old STR. All three are structurally and functionally closely linked (Alexander et al., 1986), and because of the unique anatomical properties of the STR, many studies have combined the three nuclei as STR in the past to analyze motor control and regulation functions. Few studies have separated the neostriatal caudate nucleus and putamen for functional analysis.

Neuroanatomical studies have shown that the putamen has direct anatomical connections with the M1 and SMA (Viñas-Guasch and Wu, 2017). In addition, the putamen has been shown to play an important role in motor control, with the putamen receiving voluntary motor information from corticothalamic projections for integrative processing and descending projections through ganglion brainstem networks for their characteristic motor control functions (Moustafa et al., 2018).

Moreover, fine motor control is critically involved in the transmission integration of multisensory information and is coupled with higher cognitive functions, such as learning and memory (Grosbras et al., 2011; Wacker et al., 2011; Lechak and Leber, 2012). Therefore, a thorough exploration of the neurophysiological basis underlying individual changes in motor control function after TSD is an important approach for understanding the impact of human sleep and physiological rhythms on cognitive behavior. We hypothesized that impaired fine motor control after TSD is associated with altered patterns of functional connectivity in the neostriatum, and thereby designed a 36 h TSD experiment with functional imaging data acquisition before and after sleep deprivation. The aim of this study was to identify the neurofunctional mechanisms by which sleep deprivation affects fine motor control by analyzing changes in the pattern of functional connectivity between the putamen and caudate nucleus bilaterally after TSD using resting-state fMRI.

## Materials and Methods

### Subjects

Thirty healthy adult males at university, aged 18–24 years (21.94 ± 1.73), were enrolled according to the following criteria: right handedness, normal uncorrected or corrected visual acuity, no history of alcohol and drug abuse, and no history of mental or neurological diseases. The Pittsburgh sleep quality index test scores of all subjects were less than five points, which indicated that all subjects had good sleep habits. Subjects had no history of severe physical disease or traumatic brain injury. The subjects were required not to consume alcohol, coffee, or other irritant food and drink the week before and during the experiment. The trial was approved by the Research Ethics Committee of Beihang University. The trial process and precautions were explained to the subjects before the trial. All participants voluntarily participated in the trial and provided informed consent.

### Research Methods

At the beginning of the experiment, the subjects registered their information at 16:00 on the first day and were ready to rest at 20:00. All participants underwent sleep monitoring from the first night onward, and relevant index tests was performed at 06:00 on the second day before rs-fMRI scanning including a series of emotional state scales and working memory tests. The second relevant index tests and rs-fMRI scanning were conducted at 20:00 on the third day after a 36-h sleep deprivation (SD). The participants were only allowed to perform non-strenuous activities during the 36-h period, such as conversing, reading, gaming, and working on a computer. Moreover, participants were not permitted to smoke, drink, or consume any stimulants including coffee, chocolate, soft drinks, or alcohol. Our SD laboratory used the medical sleep monitoring room in the PLA Air Force General Hospital, with complete supporting medical facilities. During the whole process of sleep deprivation, medical staff took turns on duty to ensure the health status of the subjects, and the researchers also took turns on duty to monitor the status of the subjects.

### MRI Data Acquisition

All MRI scans were conducted at the MRI Department of the PLA Air Force General Hospital. Before the scanning, the subjects were asked to take preparations (remove the magnetic items they carried, wear shoe covers, wear earplugs, etc.). The subjects lay flat on the MRI table, and their heads were fixed with sponges and bandages. Ge 3.0T MR750 equipment and a special 8-channel head coil were used to collect the MRI signals. During the scan, the subjects were asked to close their eyes and keep their head still and not think about anything and the whole procedure of the subjects in the scanner was about 40 min.

The rs-fMRI images were collected using a plane-echo imaging sequence. There were 190 images. The specific scanning parameters were as follows: repetition time, 2,000 ms; echo time, 30 ms; scanning field, 240 mm × 240 mm; layer thickness, 3 mm; layer spacing, 1 mm; turning angle, 90°; and acquisition matrix, 64 × 64. The number of layers was 35 (the scan positioning line was parallel to the anterior posterior commissural line). High-resolution T1 images were acquired using the FSPGR-BRAVO sequence. The parameters were as follows: repetition time, 8.208 s; echo time, 3.22 ms; turning angle, 12°; scanning field, 240 mm × 240 mm; inversion time, 450 ms. It is important to ensure that the subjects do not fall asleep during rs-fMRI scanning. Therefore, before each scan, they communicated with the subjects through a microphone to remind the subjects to keep awake. After each scan, subjects were asked whether they remained awake during the scan. In addition, the MRI equipment we used had a camera inside, and throughout each scan, the operator monitored the subject’s body movements and other states through the camera. Combined with their subjective reports, we can confirm that no subjects fell asleep during the scan.

### MRI Data Preprocessing

The raw MR data were analyzed using MATLAB 2015b and the statistical parametric mapping (SPM12; Welcome Department of imaging neuroscience, London, United Kingdom,^1^) software package for processing. Before resting state data preprocessing, the first 10 frames of each subject were manually removed to eliminate the effect of magnetic saturation at the initial scan stage. fMRI data preprocessing was performed next, and the specific steps mainly included slice timing, alignment, co-registration between functional and structural images, spatial normalization to MNI space (3 mm × 3 mm resolution), filtering of the waveforms of each brain voxel by band-pass filters (0.008 Hz < f < 0.09 Hz) to accommodate low-frequency drift and high-frequency noise effects, and Gaussian filter (FWHM = 6 mm) to spatially smooth the filtered data. Subjects with a head motion correction displacement of more than 2 mm in the X-, Y-, and Z-axis directions and rotation of more than 1° were removed.

### rs-fMRI Data Functional Connectivity Analysis

rs-fMRI data functional connectivity (FC) analysis was performed after preprocessing and was completed using the CONN toolbox^2^; FC analysis was performed on 30 subjects. To define the 116 regions of interest (ROIs) considered in this study, the CONN toolbox automated anatomical labeling (AAL) was used (Tzourio-Mazoyer et al., 2002), including 90 cerebral ROIs and 26 cerebellar ROIs. Linear regression analysis was performed to remove white matter, CSF, and six motor signals. The CONN toolbox extracts various subsites, that is, the average BOLD time series signals from all voxels included in the ROI region, and then summing the time series of signals from that subsite region and the correlation coefficient of the time series from each voxel of the remaining whole brain (ROI-Voxel). To estimate the intensity of FC, the correlation coefficients were converted to *Z* values using Fisher r-to-z transformation after obtaining the correlation coefficient map, resulting in an FC value for each ROI region. Paired sample *t*-tests were used to compare the differences in voxel-wise FC values between the putamen and caudate seed points to the whole brain before and after SD, with statistical significance defined as uncorrected *p* < 0.001 and cluster > 40 (Yu et al., 2020).

## Results

**FIGURE 1:**
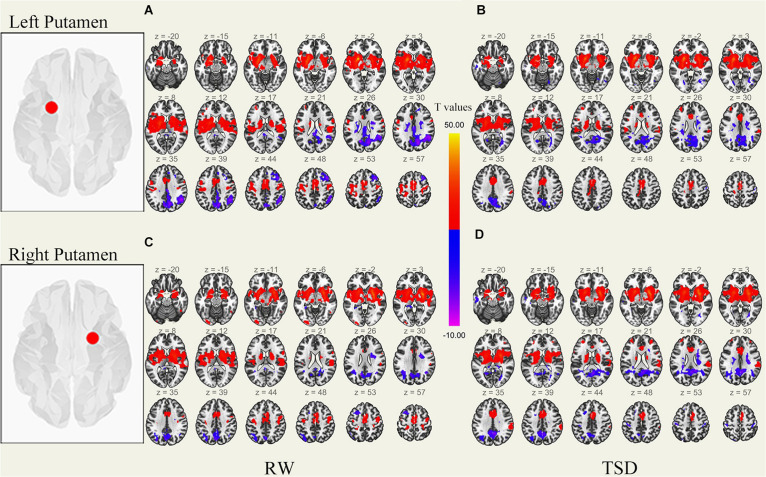
Whole-brain functional connectivity patterns of the putamen before 36 h TSD shown at panels **(A,C)**, and after 36 h TSD shown at panels **(B,D)** (*n* = 30) (Transverse view). Warm colors indicate positive correlations, and cold colors indicate negative correlations.

**FIGURE 2:**
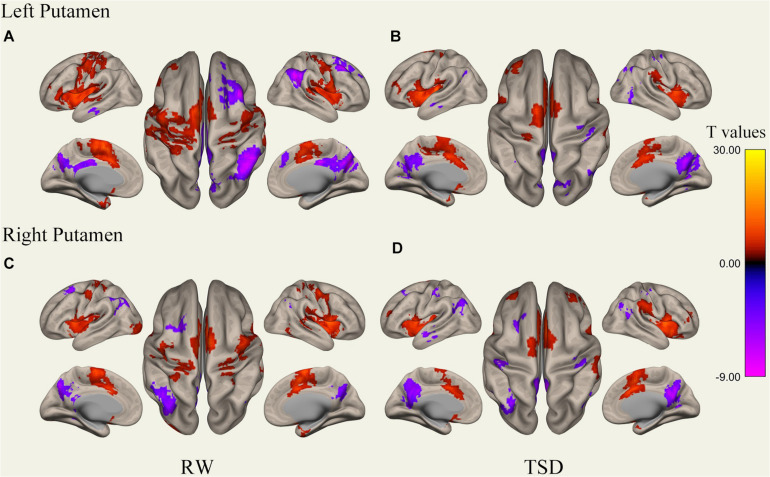
Whole-brain functional connectivity patterns of the putamen before 36 h TSD shown at panels **(A,C)**, and after 36 h TSD shown at panels **(B,D)** (*n* = 30) (Surface view). Warm colors indicate positive correlations, and cold colors indicate negative correlations.

**FIGURE 3:**
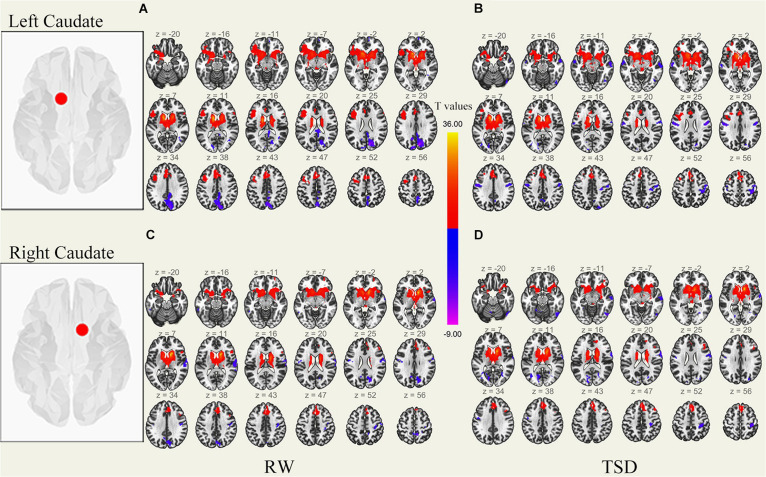
Whole-brain functional connectivity patterns of the caudate before 36 h TSD shown at panels **(A,C)**, and after 36 h TSD shown at panels **(B,D)** (*n* = 30) (Transverse view). Warm colors indicate positive correlations, and cold colors indicate negative correlations.

**FIGURE 4:**
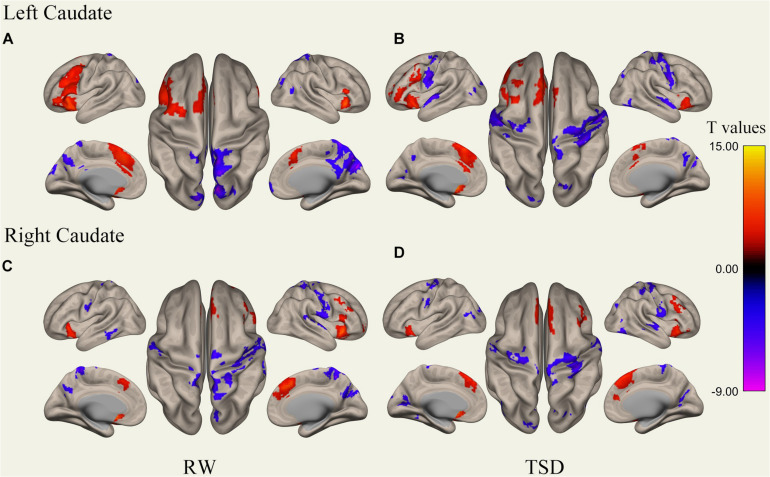
Whole-brain functional connectivity patterns of the caudate before 36 h TSD shown at panels **(A,C)**, and after 36 h TSD shown at panels **(B,D)** (*n* = 30) (Surface view). Warm colors indicate positive correlations, and cold colors indicate negative correlations.

We performed the procedure described in sections “MRI Data Preprocessing” and “rs-fMRI Data Functional Connectivity Analysis” for each subject’s data, performing correlation analysis between the BOLD signal from each ROI and whole brain voxels with paired samples *t*-tests. Results of whole-brain FC patterns in the bilateral putamen and caudate nucleus before and after sleep deprivation are detailed in Figures 1–4 and Tables in Supplementary Material.

### FC Pattern Differences in Bilateral Putamen Before and After 36 h TSD

It was found that, compared to subjects after TSD in the normal wakefulness state, the left putamen had decreased FC within the left precentral gyrus [*t*-value = −5.76, *p* < 0.001, *t*-test (GLM)], decreased FC within the right precentral gyrus [*t*-value = −5.96, *p* < 0.001, *t*-test (GLM)], and decreased FC within the left postcentral gyrus [*t*-value = −5.56, *p* < 0.001, *T*-test (GLM)], with decreased FC to the right postcentral gyrus [*t*-value = −5.50, *p* < 0.001, *t*-test (GLM)], with decreased FC to the left superior temporal gyrus [*t*-value = −6.81, *p* < 0.001, *t*-test (GLM)], and with decreased FC to the left middle temporal gyrus [*t*-value = −6.30, *p* < 0.001, *t*-test (GLM)]. However, a significant enhancement was found in the FC in the right supramarginal gyrus [*t*-value = 5.41, *p* < 0.001, *t*-test (GLM)].

**FIGURE 5:**
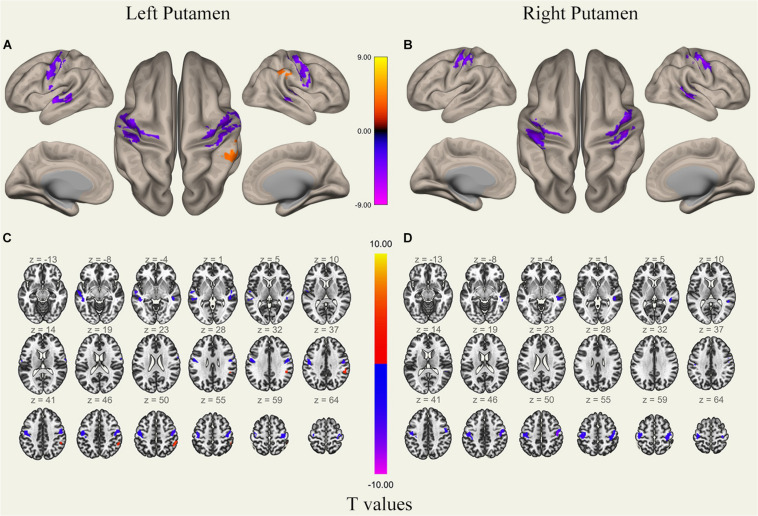
Surface views shown at panels **(A,B)** and transverse views shown at panels **(C,D)** of brain areas that exhibited altered functional connectivity with bilaterally putamen after 36 h TSD (*n* = 30). Warm colors indicate increased FC, and cold colors indicate decreased FC.

The results found that the right putamen showed similar results to the left putamen, with decreased FC between the right putamen and the left precentral gyrus after TSD [*t*-value = −5.20, *p* < 0.001, *t*-test (GLM)], decreased FC within the right precentral gyrus [*t*-value = −7.47, *p* < 0.001, *t*-test (GLM)], decreased FC within the left precentral gyrus [*t*-value = −5.73, *p* < 0.001, *T*-test (GLM)], and decreased FC within the right postcentral gyrus [*t*-value = −6.45, *p* < 0.001, *t*-test (GLM)]. However, no significant enhancement of FC was found in the right putamen (Table 1 and Figure 5).

**TABLE 1:** Changes in whole brain FC in the bilaterally Putamen before and after 36 h TSD, size of relevant regions, coordinates of MNI and maximum statistical *t* value (*n* = 30).

**Brain regions**	**Size**	**Talairach coordinates**			***T* score**
		**x**	**y**	**z**	
**Seed: Left Putamen (after TSD > before TSD)**					
Left precentral gyrus	180	−51	−3	+ 36	−5.76
Left postcentral gyrus	109	−51	−15	+ 42	−5.56
Right precentral gyrus	213	+45	−15	+ 54	−5.96
Right postcentral gyrus	120	+45	−18	+ 51	−5.50
Left superior temporal gyrus	48	−57	−12	−3	−6.81
Left middle temporal gyrus	40	−48	−36	−3	−6.30
Right supramarginal gyrus	86	+51	−42	+57	5.41
**Seed: Right Putamen (after TSD > before TSD)**					
Left precentral gyrus	108	−45	−15	+45	−5.20
Left postcentral gyrus	186	−42	−24	+57	−5.73
Right precentral gyrus	150	+45	−12	+54	−7.47
Right postcentral gyrus	106	+45	−18	+54	−6.45

### FC Pattern Differences in the Bilateral Caudate Before and After 36 h TSD

**FIGURE 6:**
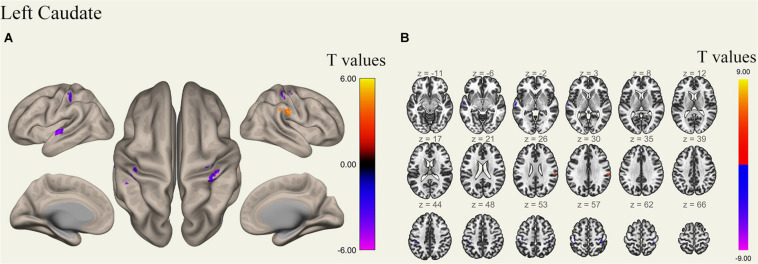
Surface views shown at panel **(A)** and transverse views shown at panel **(B)** of brain areas that exhibited altered functional connectivity with left caudate after 36 h TSD (*n* = 30). Warm colors indicate increased FC, and cold colors indicate decreased FC.

An analysis of the results only found changes in FC patterns before and after TSD in the left caudate nucleus; no significant results were found in the right caudate nucleus. Compared to subjects after TSD in the normal wakefulness state, the left caudate nucleus showed decreased FC within the left postcentral gyrus [*t*-value = −4.56, *p* < 0.001, *t*-test (GLM)], decreased FC within the right postcentral gyrus [*t*-value = −5.72, *p* < 0.001, *t*-test (GLM)], and decreased FC within the left inferior temporal gyrus [*t*-value = −6.03, *p* < 0.001, *t*-test (GLM)]. A significant enhancement was also found for FC within the right supramarginal gyrus [*t*-value = 4.82, *p* < 0.001, *t*-test (GLM)] (Table 2 and Figure 6).

**TABLE 2:** Changes in whole brain FC in the left Caudate before and after 36 h TSD, size of relevant regions, coordinates of MNI and maximum statistical *t* value (*n* = 30).

**Brain regions**	**Cluster size**	**MNI coordinates**			***T* score**
		**x**	**y**	**z**	
**Seed: Left Caudate (after TSD > before TSD)**					
Left postcentral gyrus	40	−45	−24	+54	−4.56
Right postcentral gyrus	53	+42	−30	+57	−5.72
Left inferior temporal gyrus	38	−60	−12	+03	−6.03
Right supramarginal gyrus	32	+63	−36	+33	4.82

## Discussion

In this study, we investigated the effects of 36 h TSD on whole-brain FC in the putamen and caudate nucleus of the neostriatum at resting state using fMRI. We observed a significant decrease in connectivity between the putamen and the precentral gyrus, postcentral gyrus, superior temporal gyrus, and middle temporal gyrus in subjects after TSD, as well as a significant decrease in connectivity between the left caudate and the postcentral gyrus and inferior temporal gyrus of the cortex and significantly enhanced connectivity between the left putamen and caudate with the right supramarginal gyrus. Such results could represent impaired somatic fine motor control and speech motor function due to TSD caused by disrupted communication in the motor control and regulatory network jointly involving the putamen and caudate nucleus, which is an innovative addition to the current association study of sleep loss.

By comparing putamen and caudate connectivity changes induced by loss of sleep, we found significant reductions in FC between the putamen and parts of the precentral gyrus, caudate, and parts of the postcentral gyrus. According to the cortical BG motor control loop proposed by Wall et al., the putamen and caudate nucleus receive excitatory motor information from corticocortical projections and send post-processing feedback projections back to the cortex *via* the BG (Wall et al., 2013). Thus, the reduced FC between the putamen and caudate with parietal regions after TSD compared to normal wakefulness in subjects may have contributed to the disrupted transmission of information between the neostriatum and cortical motor and sensory areas after TSD. The current study found that there is functional consolidation of motor memory by the cerebral cortical spindles during sleep, with areas of action including the hippocampus, putamen, thalamus, and somatomotor cortex (Boutin et al., 2018). TSD not only impaired this functional consolidation but also functionally separated putamen from motor control and regulatory network, which possibly causing somatic dystonia from impaired voluntary muscle control with direct consequences for individual motor control functions.

Meanwhile, the results also revealed decreased FC between the bilateral putamen and left caudate nucleus with some areas of the postcentral gyrus. This indicated that not only disturbed motor information transmission but also somatosensory information communication processes between the cerebral cortex and the neostriatum are affected by TSD in the somatomotor functions in which the BG participate. Somatosensory signals generated by the skin and proprioceptive receptors play a crucial role in the fine control of dexterous motor movements, and individuals adjust motor commands using the acquired sensory information to enable the motor system to correct sensory errors in a timely manner; the anatomical basis of this mechanism is the direct anatomical connection between the primary somatosensory cortex (Supplementary Table 1) and M1 (Mao et al., 2011; Feldmeyer, 2012). In addition, it is also a complex somatosensory motor integration function that allows quick and accurate movement (Johansson and Flanagan, 2009; Masato et al., 2019). Therefore, the reduced connectivity between the putamen, caudate, and postcentral gyrus may reflect a decline in individual fine motor control after TSD, which is associated with altered functional patterns in the sensorimotor network including the neostriatum.

However, increased FC was found between the putamen and caudate with the right supramarginal gyrus, which is considered a key region of the higher-order sensorimotor cortex and plays an important role in spatial processing and motor control. Furthermore, an fMRI study found that the right supramarginal gyrus is important for proprioception in patients with stroke (Ben-Shabat et al., 2015). The increased FC between the left putamen and left caudate with the right supramarginal gyrus in the sleep-deprived state may reflect a type of proprioceptive compensation, which, to some extent, compensates for the impaired motor control and regulatory network due to reduced connectivity in Supplementary Table 1.

It has also been found that 24-h SD causes a reduction in the density of short-distance FC in the posterior cerebellar lobes, suggesting that SD maintains cognitive performance by reducing higher-order cognition-, arousal-, and sensorimotor-related regions (Kong et al., 2018). The conclusion of this study can be similarly explained by the findings of Kong et al. that the post-TSD brain has reduced fine motor control function to maintain cognitive functional integrity by altering the neostriatal potency in motor control and regulatory network. However, the 36-h TSD paradigm used in this study, in which neuroimaging data acquisition was not performed during the procedure, only accounts for the impairment motor control and regulatory network during prolonged sleep loss and does not specifically confirm the corresponding time point of connectivity decline.

Interestingly, the putamen was found to have reduced FC in some areas of the superior and middle temporal gyrus, which we speculate is related to motor control processes related to individual speech production, simply to the movements involved in speaking. Speaking is one of the most complex and precise motor behaviors in humans; it coordinates the movements of breathing, larynx, articulation, and facial muscles to produce speech while speaking. The underlying neural mechanisms of speech involve sensory-motor interactions that incorporate feedback information for online monitoring and control of produced speech sounds. The motor behavior of speech is regulated by speech processing in areas of the auditory cortex, as demonstrated by neurophysiological studies of this sensorimotor mechanism (Flinker et al., 2010; Behroozmand and Larson, 2011; Chang et al., 2013; Greenlee et al., 2013; Sitek et al., 2013; Behroozmand et al., 2015).

An fMRI study identifying brain regions involved in the motor control of speech showed that speech feedback processing involves complex sensorimotor networks, including the superior temporal gyrus (STG), precentral gyrus, postcentral gyrus, SMA, inferior frontal gyrus (IFG), inferior parietal lobule (IPL), and insula. It has also been shown that a more complex sensory motor network involving the bilateral STG, MTG, precentral gyrus, SMA, IFG, postcentral gyrus, IPL, insula, and putamen is involved when humans use auditory feedback for speech production and motor control (Parkinson et al., 2012), which is highly consistent with our findings. From this, we speculate that speech production motor function is impaired after TSD and that this functional impairment may result from diminished connectivity of the temporal cortex speech area with the neostriatum (Ludman et al., 2000; Xu et al., 2009; Bernstein et al., 2011; Wu et al., 2017).

Our study has some limitations. First, we only assessed male volunteers, so we cannot make generalizations to females. Only male volunteers were recruited due to the experimental conditions and the long time course of the study. This will limit the clinical utility of the findings. In the future, it would be interesting to investigate sex differences in functional connectivity changes following TSD.

Second, circadian biorhythms affect behavioral performance, and these effects differ across individuals (Montplaisir, 1981; Lavie, 2001). However, considering that 48 h of sleep deprivation may uncontrollably damage the health of the subjects and 24 h of sleep deprivation has relatively small effects, 36 h of sleep deprivation is inevitably affected by circadian rhythm. In previous similar studies, the data collected at 20:00 on the day before sleep deprivation and 08:00 on the first day were used as two baselines, but the results showed that there was no significant difference between the two baselines (Shao et al., 2014; Lei et al., 2015; Zhang et al., 2019; Peng et al., 2020; Li et al., 2021); therefore, only one baseline was collected in this study. However, no circadian/time of day differences in other brain functions does not mean that neostriatum connectivity is unaffected by circadian/time of day effects. Thus, we could not rule out effects of circadian rhythms on our results, and in future experiments, we will consider measuring two baseline data and using the Horne and Ostberg questionnaire to determine the chronotype of the subject to exclude the influence of circadian rhythm.

Third, although behavioral assessment was used to evaluate sleep during MRI scanning, this method may not be completely sufficient. Fluctuation in states of sleepiness, drowsiness, and sudden (even if very short) sleep episodes of a few seconds, cannot be 100% excluded, which is a major limitation of the study. Polysomnography, which is the gold standard for sleep evaluation, and other physiological monitoring methods lacking here, such as long-term EEG or ECG, should be considered in future research.

## Conclusion

Overall, reduced connectivity between the putamen and the precentral gyrus leads to a blockade of the cortico BG motor control circuit and impaired motor control and regulatory network in individuals. In addition, connectivity between the putamen and caudate nucleus with the postcentral gyrus leads to disrupted sensory information feedback in the somatosensory motor integration system, which in turn affects individual fine motor control function. Decreased connectivity of the putamen with the STG and MTG and the caudate with the ITG may lead to impaired speech production and speech motor control in individuals due to the separation of speech and sensorimotor information in the speech sensory motor network.

## Data Availability Statement

The raw data supporting the conclusions of this article will be made available by the authors, without undue reservation.

## Ethics Statement

The studies involving human participants were reviewed and approved by Research Ethics Committee of Beihang University. The patients/participants provided their written informed consent to participate in this study.

## Author Contributions

YS and WF designed the study. HW and KY conceptualized, investigated, and visualized the data, carried out the formal analysis, and wrote the manuscript. TY, LZ, JL, CD, and ZP contributed to the data collection. YS and JQ were the guarantors of this study. All authors contributed to the article and approved the submitted version.

## Conflict of Interest

The authors declare that the research was conducted in the absence of any commercial or financial relationships that could be construed as a potential conflict of interest.

## Publisher’s Note

All claims expressed in this article are solely those of the authors and do not necessarily represent those of their affiliated organizations, or those of the publisher, the editors and the reviewers. Any product that may be evaluated in this article, or claim that may be made by its manufacturer, is not guaranteed or endorsed by the publisher.

## Footnotes

1
http://www.fil.ion.ucl.ac.uk/spm/software/spm12/

2
http://web.mit.edu/swg/software.htm
