# Supplementary material for: Altered Functional Connectivity in the Resting State Neostriatum After Complete Sleep Deprivation: Impairment of Motor Control and Regulatory Network
Source: Front Neurosci. 2021 Aug 17;15:665687. doi: 10.3389/fnins.2021.665687 (PMC8416068; doi:10.3389/fnins.2021.665687)
Supplement: Supplementary file 1 [file Table_1.DOCX]

**Table** FC of bilateral putamen in normal arousal and TSD state, size of relevant areas, MNI coordinates and maximum statistical T values (n = 30)

| **Brain regions** | **Cluster Size** | **MNI coordinates** | | | **T score** |
| --- | --- | --- | --- | --- | --- |
|  |  | **x** | **y** | **z** |  |
| **Left Putamen connectivity in RW** |  |  |  |  |  |
| Precentral Gyrus Left | 629 | -34 | -12 | 49 | 4.35 |
| Precuneous Cortex | 578 | 1 | -59 | 38 | -4.11 |
| Precentral Gyrus Right | 428 | 39 | -15 | 45 | 4.90 |
| Insular Cortex Left | 360 | -36 | 1 | 0 | 10.66 |
| Postcentral Gyrus Left | 332 | -38 | -28 | 52 | 4.30 |
| Cingulate Gyrus, posterior division | 302 | 1 | -37 | 30 | -4.33 |
| Middle Frontal Gyrus Right | 269 | 39 | 19 | 43 | -7.44 |
| Angular Gyrus Right | 257 | 52 | -52 | 32 | -9.88 |
| Insular Cortex Right | 248 | 37 | 3 | 0 | 6.63 |
| Central Opercular Cortex Left | 246 | -48 | -9 | 12 | 6.71 |
| Thalamus l | 245 | -10 | -19 | 6 | 6.67 |
| Central Opercular Cortex Right | 208 | 48 | -18 | 18 | 6.37 |
| Cingulate Gyrus, anterior division | 200 | -6 | 15 | 33 | 8.46 |
| Juxtapositional Lobule Cortex Left | 168 | -5 | -3 | 56 | 9.82 |
| Lateral Occipital Cortex, superior division Right | 154 | 42 | -60 | 42 | -6.75 |
| Caudate l | 143 | -13 | 9 | 10 | 10.64 |
| Thalamus r | 139 | 15 | -15 | 6 | 7.78 |
| Juxtapositional Lobule Cortex Right | 137 | 6 | -3 | 58 | 6.62 |
| Frontal Orbital Cortex Left | 113 | 45 | 18 | -9 | 5.61 |
| Postcentral Gyrus Right | 108 | 42 | -18 | 48 | 4.56 |
| Temporal Pole Left | 107 | -40 | 12 | -9 | 5.85 |
| Amygdala l | 93 | -23 | -5 | -18 | 12.79 |
| Caudate r | 80 | 18 | 12 | 12 | 11.86 |
| Pallidum l | 71 | -19 | -5 | -1 | 13.60 |
| Frontal Operculum Cortex Left | 59 | -42 | 9 | 6 | 7.48 |
| Paracingulate Gyrus Left | 55 | -3 | 9 | 48 | 9.62 |
| Hippocampus l | 53 | -30 | -15 | -15 | 5.76 |
| Parahippocampal Gyrus, anterior division Left | 50 | -18 | -3 | -24 | 8.52 |
| Middle Frontal Gyrus Left | 48 | -51 | 9 | 36 | 5.87 |
| Pallidum r | 48 | 24 | -6 | 3 | 12.51 |
| **Left Putamen connectivity in TSD** |  |  |  |  |  |
| Precuneous Cortex | 544 | 6 | -57 | 33 | -7.02 |
| Insular Cortex Left | 338 | -36 | 10 | 0 | 6.85 |
| Cingulate Gyrus, anterior division | 335 | 3 | 18 | 27 | 6.97 |
| Insular Cortex Right | 276 | 37 | 11 | 0 | 5.65 |
| Thalamus l | 232 | -9 | 0 | 6 | 7.53 |
| Thalamus r | 185 | 9 | -3 | 6 | 8.27 |
| Cingulate Gyrus, posterior division | 140 | -9 | -45 | 30 | -5.31 |
| Caudate l | 137 | -13 | -3 | 10 | 9.40 |
| Juxtapositional Lobule Cortex -formerly Supplementary Motor Cortex- Left | 128 | -5 | -3 | 56 | 4.43 |
| Frontal Pole Left | 124 | -25 | 5 | 8 | 17.66 |
| Juxtapositional Lobule Cortex -formerly Supplementary Motor Cortex- Right | 120 | 3 | 6 | 48 | 8.36 |
| Central Opercular Cortex Left | 107 | -48 | -1 | 12 | 5.57 |
| Frontal Orbital Cortex Left | 101 | 24 | 9 | -18 | 7.70 |
| Amygdala l | 90 | -23 | -5 | -18 | 14.88 |
| Cuneal Cortex Right | 88 | 15 | -78 | 24 | -5.12 |
| Caudate r | 87 | 13 | -2 | 10 | 9.73 |
| Precentral Gyrus Left | 76 | -34 | -24 | 57 | 5.20 |
| Pallidum l | 75 | -19 | 14 | -1 | 22.65 |
| Superior Frontal Gyrus Left | 68 | -12 | -3 | 69 | 7.86 |
| Pallidum r | 59 | 20 | 15 | -1 | 13.47 |
| Paracingulate Gyrus Right | 50 | 7 | 17 | 23 | 7.61 |
| Hippocampus l | 49 | -18 | -12 | -18 | 7.68 |
| Temporal Pole Left | 47 | -40 | 23 | -30 | 5.27 |
| Frontal Orbital Cortex Right | 46 | 29 | 4 | -16 | 12.54 |
| Heschl's Gyrus Left | 45 | -45 | 6 | 7 | 6.63 |
| Hippocampus r | 43 | 30 | -18 | -12 | 6.47 |
| Amygdala r | 42 | 27 | 0 | -15 | 15.90 |
| Paracingulate Gyrus Left | 42 | -6 | 16 | 21 | 7.22 |
| **Right Putamen connectivity in RW** |  |  |  |  |  |
| Precuneous Cortex | 435 | 1 | -59 | 38 | -4.56 |
| Precentral Gyrus Right | 325 | 45 | 0 | 45 | 7.10 |
| Insular Cortex Right | 274 | 37 | 3 | 0 | 10.28 |
| Precentral Gyrus Left | 253 | -39 | -15 | 45 | 6.63 |
| Insular Cortex Left | 246 | -36 | 1 | 0 | 8.26 |
| Central Opercular Cortex Left | 213 | -48 | -9 | 12 | 4.63 |
| Central Opercular Cortex Right | 209 | 49 | -6 | 11 | 4.25 |
| Lateral Occipital Cortex, superior division Left | 206 | -32 | -73 | 38 | -5.72 |
| Cingulate Gyrus, anterior division | 203 | 0 | 9 | 42 | 8.70 |
| Thalamus r | 184 | 6 | -3 | 3 | 7.84 |
| Juxtapositional Lobule Cortex -formerly Supplementary Motor Cortex- Right | 159 | 6 | -3 | 58 | 8.41 |
| Thalamus l | 140 | -9 | -24 | 0 | 6.61 |
| Juxtapositional Lobule Cortex -formerly Supplementary Motor Cortex- Left | 132 | -5 | -3 | 61 | 6.67 |
| Caudate l | 121 | -13 | 9 | 10 | 7.45 |
| Caudate r | 114 | 13 | 10 | 10 | 10.89 |
| Postcentral Gyrus Left | 90 | -27 | -39 | 57 | 5.96 |
| Amygdala r | 88 | 23 | -4 | -18 | 6.51 |
| Amygdala l | 86 | -23 | -5 | -18 | 9.97 |
| Occipital Pole Left | 85 | -27 | -97 | -3 | 5.69 |
| Pallidum l | 68 | -19 | -5 | -1 | 11.24 |
| Superior Frontal Gyrus Left | 61 | -24 | 15 | 54 | -5.75 |
| Postcentral Gyrus Right | 59 | 27 | -33 | 57 | 6.06 |
| Inferior Frontal Gyrus, pars opercularis Right | 58 | 57 | 12 | 18 | 6.52 |
| Lateral Occipital Cortex, inferior division Left | 58 | -33 | -87 | -12 | 6.02 |
| Pallidum r | 56 | 24 | -6 | 0 | 15.20 |
| Superior Frontal Gyrus Right | 55 | 12 | -3 | 66 | 6.81 |
| Cingulate Gyrus, posterior division | 52 | -3 | -42 | 18 | -5.58 |
| Paracingulate Gyrus Right | 50 | 3 | 9 | 48 | 8.98 |
| Middle Frontal Gyrus Left | 50 | -33 | 15 | 57 | -6.40 |
| Frontal Operculum Cortex Right | 47 | 41 | 19 | 5 | 5.86 |
| Planum Polare Right | 47 | 54 | 9 | -3 | 7.30 |
| Temporal Pole Right | 43 | 27 | 6 | -21 | 6.83 |
| Frontal Operculum Cortex Left | 36 | -40 | 18 | 5 | 5.87 |
| Hippocampus r | 36 | 30 | -12 | -15 | 7.49 |
| Accumbens l | 34 | -9 | 11 | -7 | 5.18 |
| **Right Putamen connectivity in TSD** |  |  |  |  |  |
| Precuneous Cortex | 553 | 6 | -57 | 33 | -6.60 |
| Cingulate Gyrus, anterior division | 336 | 3 | 18 | 27 | 7.63 |
| Insular Cortex Left | 305 | -36 | 10 | 0 | 7.04 |
| Insular Cortex Right | 299 | 37 | 11 | 0 | 7.23 |
| Thalamus r | 197 | 9 | -3 | 6 | 9.40 |
| Thalamus l | 185 | -9 | 0 | 6 | 6.50 |
| Lateral Occipital Cortex, superior division Left | 183 | -30 | -66 | 42 | -5.42 |
| Cingulate Gyrus, posterior division | 178 | -9 | -45 | 30 | -5.76 |
| Frontal Orbital Cortex Right | 160 | 24 | 9 | -18 | 8.89 |
| Caudate l | 138 | -13 | -3 | 10 | 9.43 |
| Juxtapositional Lobule Cortex -formerly Supplementary Motor Cortex- Right | 127 | 3 | 6 | 48 | 9.82 |
| Supramarginal Gyrus, anterior division Right | 123 | 57 | -27 | 36 | 5.65 |
| Caudate r | 109 | 13 | -2 | 10 | 13.60 |
| Paracingulate Gyrus Right | 81 | 6 | 9 | 48 | 9.41 |
| Postcentral Gyrus Left | 78 | -48 | -21 | 54 | -6.35 |
| Pallidum l | 77 | -19 | 14 | -1 | 16.33 |
| Frontal Orbital Cortex Left | 73 | -29 | 4 | -16 | 6.87 |
| Central Opercular Cortex Left | 72 | -49 | 0 | 11 | 5.99 |
| Parietal Operculum Cortex Right | 71 | 57 | -30 | 21 | 5.58 |
| Amygdala l | 69 | -23 | -5 | -18 | 10.08 |
| Pallidum r | 65 | 20 | 15 | -1 | 18.59 |
| Inferior Frontal Gyrus, pars opercularis Right | 64 | 51 | 9 | 21 | 5.98 |
| Temporal Pole Right | 63 | 27 | 6 | -21 | 6.51 |
| Frontal Operculum Cortex Right | 61 | 38 | 15 | 5 | 6.26 |
| Juxtapositional Lobule Cortex -formerly Supplementary Motor Cortex- Left | 60 | -3 | 6 | 48 | 5.97 |
| Amygdala r | 60 | 27 | 0 | -15 | 19.57 |
| Angular Gyrus Left | 52 | -45 | -60 | 30 | -5.31 |
| Angular Gyrus Right | 49 | 51 | -57 | 15 | -5.73 |
| Superior Frontal Gyrus Left | 45 | -12 | -3 | 69 | 5.44 |
