# Supplementary material for: Altered Functional Connectivity in the Resting State Neostriatum After Complete Sleep Deprivation: Impairment of Motor Control and Regulatory Network
Source: Front Neurosci. 2021 Aug 17;15:665687. doi: 10.3389/fnins.2021.665687 (PMC8416068; doi:10.3389/fnins.2021.665687)
Supplement: Supplementary file 2 [file Table_2.DOCX]

**Table** FC of bilateral caudate in normal arousal and TSD state, size of relevant areas, MNI coordinates and maximum statistical T values (n = 30)

| **Brain regions** | **Cluster Size** | **MNI coordinates** | | | **T score** |
| --- | --- | --- | --- | --- | --- |
|  |  | **x** | **y** | **z** |  |
| **Left Caudate connectivity in RW** |  |  |  |  |  |
| Precuneous Cortex | 577 | 6 | -78 | 39 | -6.71 |
| Middle Frontal Gyrus Left | 400 | -42 | 3 | 48 | 5.90 |
| Inferior Frontal Gyrus left | 288 | -48 | 27 | 15 | 6.84 |
| Frontal Orbital Cortex Left | 284 | -33 | 21 | -12 | 8.02 |
| Thalamus Left | 227 | -12 | -3 | 12 | 18.28 |
| Putamen Left | 193 | -18 | 18 | -3 | 18.67 |
| Superior Frontal Gyrus Left | 174 | -6 | 24 | 48 | 6.83 |
| Putamen Right | 156 | 18 | 12 | 0 | 15.76 |
| Paracingulate Gyrus Left | 146 | -3 | 27 | 39 | 7.30 |
| Posterior Cingulate Gyrus | 140 | 6 | -48 | 36 | -4.97 |
| Thalamus Right | 134 | 6 | -3 | 6 | 11.95 |
| Cuneal Cortex Right | 130 | 9 | -78 | 30 | -8.54 |
| Lateral Occipital Cortex Right | 114 | 21 | -81 | 27 | -5.88 |
| Frontal Orbital Cortex Right | 106 | 30 | 24 | -6 | 9.41 |
| Frontal Pole Left | 99 | -36 | 42 | -9 | 7.59 |
| Insular Cortex Left | 69 | -27 | 24 | -3 | 8.16 |
| Paracingulate Gyrus Right | 66 | 3 | 21 | 48 | 6.92 |
| **Left Caudate connectivity in TSD** |  |  |  |  |  |
| Precentral Gyrus Right | 326 | 30 | -24 | 63 | -5.54 |
| Thalamus Left | 265 | -12 | -2 | 12 | 17.97 |
| Frontal Orbital Cortex Left | 235 | -43 | 21 | -12 | 8.18 |
| Putamen Right | 211 | -18 | 15 | -6 | 14.86 |
| Superior Frontal Gyrus Left | 207 | -6 | 24 | 51 | 6.76 |
| Putamen Left | 202 | 18 | 15 | -3 | 15.99 |
| Thalamus Right | 196 | 9 | 0 | 9 | 17.89 |
| Postcentral Gyrus Right | 194 | 42 | -30 | 57 | -6.45 |
| Middle Frontal Gyrus Left | 155 | -30 | 27 | 30 | 5.97 |
| Precentral Gyrus Left | 121 | -51 | -6 | 36 | -6.08 |
| Frontal Orbital Cortex Right | 117 | -42 | 21 | -12 | 8.18 |
| Paracingulate Gyrus Left | 116 | -3 | 27 | 33 | 7.52 |
| Anterior Cingulate Gyrus | 87 | -3 | 24 | 30 | 5.77 |
| Frontal Pole Left | 79 | -39 | 39 | 6 | 6.05 |
| Postcentral Gyrus Left | 75 | -48 | -12 | 30 | -6.11 |
| Right Caudate connectivity in RW |  |  |  |  |  |
| Precuneous Cortex | 259 | 3 | -75 | 39 | -5.16 |
| Postcentral Gyrus Right | 228 | 60 | -6 | 14 | -5.67 |
| Thalamus r | 170 | 12 | -6 | 15 | 17.87 |
| Putamen r | 164 | 21 | 18 | 0 | 14.5 |
| Frontal Orbital Cortex Right | 162 | 27 | 21 | -9 | 10.21 |
| Thalamus l | 151 | -3 | -3 | 3 | 12.05 |
| Putamen l | 129 | -18 | 18 | -3 | 11.13 |
| Frontal Orbital Cortex Left | 127 | -30 | 27 | -6 | 6.49 |
| Paracingulate Gyrus Right | 119 | 3 | 33 | 39 | 9.37 |
| Precentral Gyrus Right | 109 | 39 | -12 | 45 | -5.13 |
| Superior Frontal Gyrus Right | 106 | 6 | 39 | 39 | 7.28 |
| Postcentral Gyrus Left | 79 | -57 | -12 | 24 | -4.74 |
| Cuneal Cortex Right | 64 | 18 | -72 | 27 | -5.96 |
| Planum Temporale Right | 53 | 60 | -24 | 12 | -5.43 |
| Right Caudate connectivity in TSD |  |  |  |  |  |
| Precentral Gyrus Right | 230 | 63 | 3 | 21 | -5.27 |
| Thalamus l | 210 | -6 | -3 | 6 | 13.33 |
| Thalamus r | 206 | 9 | 0 | 9 | 17.64 |
| Putamen r | 192 | 15 | 9 | -3 | 12.36 |
| Postcentral Gyrus Right | 178 | 66 | -3 | 21 | -4.95 |
| Frontal Orbital Cortex Right | 163 | 39 | 24 | -15 | 8.13 |
| Superior Frontal Gyrus Right | 154 | 3 | 42 | 42 | 7.19 |
| Putamen l | 153 | -21 | 9 | 0 | 9.03 |
| Lateral Occipital Cortex Right | 115 | 51 | -72 | -12 | -5.47 |
| Superior Frontal Gyrus Left | 102 | -3 | 36 | 48 | 7.19 |
| Frontal Orbital Cortex Left | 100 | -33 | 24 | -9 | 5.34 |
| Precentral Gyrus Left | 99 | -51 | -6 | 36 | -4.68 |
| Middle Frontal Gyrus Right | 91 | 42 | 18 | 30 | 6.21 |
| Paracingulate Gyrus Right | 74 | 12 | 42 | 18 | 5.12 |
